# Recent updates in the indolent lymphomas: Update on marginal zone lymphoma and Waldenström's macroglobulinemia

**DOI:** 10.1002/hon.3210

**Published:** 2023-07-17

**Authors:** Karima Amaador, Catherine Thieblemont, Judith Trotman, Monique C. Minnema

**Affiliations:** ^1^ Department of Internal Medicine UMC Utrecht Utrecht The Netherlands; ^2^ Université Paris Cité Assistance Publique & Hôpitaux de Paris Paris France; ^3^ Department of Haematology Concord Repatriation General Hospital and Faculty of Medicine and Health University of Sydney Concord New South Wales Australia; ^4^ Department of Hematology UMC Utrecht University Utrecht Utrecht The Netherlands

**Keywords:** marginal zone lymphoma, treatment, Waldenstrom macroglobulinemia

## Abstract

Marginal Zone Lymphoma (MZL) and Waldenström's Macroglobulinemia (WM) are indolent lymphomas that both arise from post germinal center lymphocytes. Both can secrete a monoclonal protein but high levels are mostly only seen in WM. The MYD88 L256P somatic mutation that is present in an estimated 95% of patients with WM has helped greatly in differentiating the two lymphomas. Several large clinical studies with new drugs have been performed that have provided new treatment options for both MZL and WM patients. In this short review we will discuss the recent literature published and provide some recommendations.

## 1 MARGINAL ZONE LYMPHOMA

**Epidemiology**. Marginal zone lymphomas (MZL) are indolent mature B‐cell lymphomas, with variable epidemiologic, pathologic, and clinical features. Three main subtypes described are extranodal MZL (Extranodal marginal zone lymphoma [EMZL]), splenic MZL (SMZL), and nodal MZL (NMZL).^1^ It represents 7% of all mature non‐Hodgkin lymphomas in the United States.^2^ Based on data from the US SEER‐18 program from 2001 to 2017, the age‐standardized incidence rate for MZL was 19.6 per 1,000,000 person‐years; 9% of MZL cases were SMZL, 30% NMZL, and 61% extranodal MZL (EMZL) of mucosa‐associated lymphoid tissue (MALT).^2^ EMZL presents as a localized disease in most cases, often arising in response to various infectious, autoimmune, or other inflammatory stimuli, which leads to a neoplastic transformation. Typically, gastric EMZL is related to *Helicobacter pylori*, the paradigm for the association between tumorigenesis and a chronic inflammatory stimulus. However, this association seems to decrease over the past 20 years, from 61% to 17% of patients diagnosed before and after 2002 in northern Italy, with an impact on the incidence of gastric EMZL dropping from 1.4 in 1997 to 0.2 in 2002, probably caused by a decreased rate of HP infection among the healthy population.^3^ The sites involved may arise either from mucosa such as in stomach, intestine, lungs, thyroid, salivary gland (MALT lymphoma), or from non‐mucosal sites such as meninges, skin, and orbit. The common extranodal sites include the stomach (30%), ocular adnexal (OA; 12%), skin (10%), lung (9%), and salivary gland (7%).^2^ Patients with SMZL present with splenomegaly, bone marrow (BM) and usually blood involvement. Anemia and thrombocytopenia may be present. While splenic hilar nodes can be involved, more distant nodal disease is rare. Nodal MZL usually presents as a disseminated nodal disease without evidence of extranodal or splenic involvement. The most recent WHO classification defined cutaneous MZL lymphoma as a separate entity because of its different and even more indolent behavior and specific mutations such as FAS, SLAMF1, SPEN, and NCOR2 not seen in other MZL.^4^, ^5^, ^6^ Also pediatric NMZL was added as a provisional entity.^5^

**Biology and molecular findings**. A number of clinical, biological, and pathological studies specifically devoted to MZL has confirmed the unique biology of the disease(s). No ancillary biomarker exist. Deletion 7q22‐36 (seen in 30%–40% of cases of SMZL), mutations in KLF2 (12%–42%) and NOTCH2 (10%–25%) are frequently seen.^7^, ^8^, ^9^, ^10^ Nonmutated IGHV as well as mutations in NOTCH2, KLF2, and TP53 have been associated with inferior outcomes.^7^, ^11^, ^12^ Mutations in MYD88 L265P can be also observed in 7%–15% of SMZL cases.

**PET/CT in the management of MZL**. Among the imaging testing aimed at assessing disease dissemination, the use of PET/CT in MZL staging and response assessment is being implemented more and more in routine MZL assessment, even if Lugano classification originally listed MZLs as non‐fluorodeoxyglucose (FDG)‐avid diseases without recommending PET/CT.^13^ PET/CT is able to detect BM involvement in MZL in only around one‐third of cases: this implies that BM biopsy is still needed, if relevant, for therapeutic decisions.^14^ However, a recent retrospective study showed that BM biopsy may not be required because it does not affect lymphoma‐related outcomes in patients with clinically/imaging‐based localized EMZL treated with radiotherapy (RT).^15^

**Prognostic scores**. The MALT International Prognostic Index can be used for the prognostic stratification of extranodal MZLs.^16^ The HPLL score (named for the factors used to calculate the score, including hemoglobin, platelet count, lactate dehydrogenase, and extra hilar lymphadenopathy), can be used for the prognostic stratification of splenic MZLs.^17^ Age at presentation is the only consistent prognostic factor for nodal MZLs, since pediatric cases have an excellent prognosis.^5^

**Therapeutic strategies**. MZL has a natural indolent course, with many patients surviving beyond 10 years from diagnosis.^1^ However, in approximately 20% of MZL patients, who relapse or progress within 2 years, median overall survival (OS) is only 3–5 years.^18^, ^19^, ^20^

Optimal treatment for MZL is not clearly standardized.^21^ At diagnosis, a watch and wait strategy can be considered. Based on expert opinion, therapy initiation may be proposed when patients present with lymphoma‐related symptoms, gastrointestinal bleeding, deep organ invasion, threatened end‐organ function, bulky disease, and rapid disease progression; patient preference must be considered as well.^22^

**First‐line local treatment**. A pathogen‐directed therapy in case of association with microbial pathogens can induce long‐term control particularly in Helicobacter pylori‐associated gastric EMZL. If no association has been diagnosed, the first‐line treatment option will consider the type of entities (EMZL, SMZL or NMZL) and the extension of the disease. Local therapy such as surgery, may play a role in the treatment of isolated tumors that are not amenable to RT because of their location (e.g., in the lung) or in superficial anatomical sites, or use of involved‐site radiation therapy with a dosing between 20 and 30 Gy in locations such as orbit, skin, or t(11;18) negative gastric EMZL.^23^, ^24^, ^25^ In SMZL, splenectomy should be reserved for those with symptomatic massive SMZL refractory to first line therapy.

**First‐line systemic treatment**. Systemic therapy is based on chemoimmunotherapy rituximab‐based approaches for symptomatic, advanced‐stage MZL, except SMZL where single agent rituximab is proposed.^26^, ^27^ The chemotherapy backbone (chlorambucil or bendamustine) is chosen according to the patient's age and status with respect to fitness and organ functions, since older or frail patients receiving Bendamustine‐Rituximab (BR) may have high rates of fatal toxic effects.^27^, ^28^, ^29^, ^30^, ^31^ In this later case, chlorambucil could be considered for patients with EMZL who require systemic therapy but are not deemed candidates for BR.

**Novel therapies in relapsed/refractory MZLs**. Bruton Tyrosine Kinase (BTK) inhibition has been proven effective in patients with relapsed MZL with response rates between 53% and 68% in several prospective phase II trials with ibrutinib (NCT01980628), acalabrutinib (NCT02180711), and zanubrutinib (NCT03846427), respectively.^32^, ^33^, ^34^, ^35^ Median duration of response has been 27.6 months in the ibrutinib trial and not reached in the others due to a shorter follow up (FU) period. Although ibrutinib received FDA accelerated approval specifically for MZL, recently its MZL indication was voluntarily withdrawn from the market in the US due to inability to meet sufficient primary endpoint (Progression Free Survival [PFS]) in confirmatory phase III study (SELENE NCT01974440). Moreover, ibrutinib appears to have inferior tolerability and efficacy compared to next‐generation BTKi's such as zanubrutinib (which maintains its FDA indication). Lenalidomide with rituximab (R2) is an active combination in MZL, currently FDA approved after 1 line of therapy. The AUGMENT trial (NCT01938001) compared R2 versus Rituximab + placebo in patients with relapsed/refractory follicular lymphoma or MZL.^36^ The sub‐analysis specific to patients with MZL (*n* = 63; 18%) including patients with EMZL (*n* = 30), NMZL (*n* = 18), or SMZL (*n* = 15), showed an overall response rates (ORR) of 78% (CR, 34%) versus 53% (CR, 18%; *P* = 0.001) and also the primary study end point of PFS (HR, 0.46; *P* < 0.0001) favored R2. None of the phosphatidylinositol 3‐kinase (PI3K) inhibitors (Idelalisib, Copanlisib, Parsaclisib, Umbralisib) analyzed in R/R MZL are available for patients, safety being a major issue.^37^, ^38^, ^39^, ^40^ Recently, ZUMA 5 (NCT03105336) assessed the efficacy of axicabtagene ciloleucel, an autologous anti‐CD19 chimeric antigen receptor (CAR) T‐cell therapy in relapsed or refractory indolent non‐Hodgkin lymphoma including 24 (16%) MZL.^41^ In this specific cohort of MZL, the ORR was 83% including a CR rate at 63%. The 24‐month PFS and OS were at 47.4% (95% CI, 23.1–68.4) and 69.9% (95% CI, 44.0–85.5), respectively. Notably, anti‐CD19 CAR T cell therapy has been also assessed in a cohort of transformed MZL patients in a single center retrospective study.^42^ The CR rate was 71.4% and median PFS was not reached with a median follow‐up of 21.3 months.

**In conclusion**, the current therapeutic landscape in MZL allows clinicians to limit the use of cytotoxic chemo‐therapy. Access to novel therapies remains challenging for MZL because of relative rarity of MZL subtypes with different biology and clinical behavior and thus distinct indications for and response to a given therapy. In addition, there are difficulties in applying typical lymphoma diagnostic/response criteria (e.g., PET) in EMZL and SMZL, which often results in exclusion of these cases from studies not specifically designed for MZL. The future should help to delineate the best therapeutic sequence for patients with MZL.

## 2 WALDENSTRÖM's MACROGLOBULINEMIA

### 2.1 Biology and molecular findings

Waldenström's Macroglobulinemia (WM) is an atypical lymphoma given its common presentation with predominant extra nodal involvement with obligatory infiltration of the BM. Secondly, due to the presence of not only clonal B cells but also clonal plasma cells, this lymphoma secretes high levels of an IgM monoclonal protein (M‐protein). WM is defined by the presence of both the lymphoplasmacytic lymphoma (LPL) and the IgM M protein; as such, LPL that secretes an IgG or IgA M protein (<5% of LPL) is therefore not WM.^43^

Important insights in biology have been achieved by the finding of a MYD88 L256P somatic mutation in more than 95% of patients, as well as CXCR4 mutations in almost 40% of patients. While the mutational status of both *MYD88* and *CXCR4* is ideally determined in all patients, technical challenges in identifying *CXCR4* mutations and the relative impact of different *CXCR4* mutations prevent widespread testing in clinical practice. While almost all CXCR4 mutations impair CXCR4 internalization, patients with frameshift mutations can have a major response with Ibrutinib therapy, while patients with a nonsense mutation seem to have a worse response to this drug.^44^ A clinical guideline from the European Consortium for Waldenström's Macroglobulinemia (ECWM) updating all recent diagnostic information is published and the diagnostic consensus guidelines from the 2022 International Workshop in WM are also published.^45^, ^46^ The ECWM guideline focusses on the laboratory diagnosis and provides practical advice on standardized multiparametric flow cytometry protocols, how to handle MYD88 negative PCR results and further molecular testing after CD19 selection of BM aspirates. The guidelines form the International Workshop have simplified the response assessments in using serum IgM only and not extramedullary diseases assessment for determining partial and very good partial responses, since these simplified criteria maintained the same prognostic PFS criteria as the previous response criteria.^46^, ^47^ In addition, re‐confirmation of response with a second serum IgM measurement is not required.

WM patients can have multiple relapses during their disease course. Applying whole genome sequencing demonstrated that genomic instability such as increased copy number aberrations tend to occur more frequently in relapsed disease.^48^ An analysis using the nationwide Netherlands Cancer Registry demonstrated that contemporary diagnosed WM patients, compared to the general population, continue to experience gradual increase in excess mortality with each additional year survived post diagnosis.^49^ This is likely due to the incurable nature of WM characterized by multiple relapses, combined with this increased genomic instability, increased risk of other cancers and limited treatment options in the released/refractory setting.

An interesting new concept for clinical research is the first global patient driven WhiMSICAL registry, which identified marked global variation in WM treatment.^50^ Analysis of the data entered by 302 participants who had received treatment identified 46 unique first‐line therapies after combining all clinical trial treatments. This variation was maintained across countries, although higher in the USA with 36 unique therapies used (*n* = 136) and 27 in the non‐USA population (*n* = 166). In another study, patients opinion on treatment was analyzed with a discrete choice experiment. In such an experiment, patients receive several questions in which tradeoffs are made to assess relative importance of certain characteristics of treatments. This can be for example, fixed versus continuous treatment, oral versus intravenous treatment, etc. Answers of 214 patients demonstrated that treatment efficacy was the most important attribute for patients, followed by a low risk of future secondary malignancies.^51^

**First‐line systemic treatment**. Dexamethasone, cyclophosphamide and rituximab (DRC) and BR therapy are commonly used. There is considerable debate over whether 6 cycles of full dose BR with 90 mg/m^2^ bendamustine on days 1 and 2 is necessary in WM treatment. In a recent multicenter, retrospective cohort analysis of 250 WM patients treated with BR in the first‐line or relapsed settings, total bendamustine dose impacted response and PFS.^52^ In the first‐line setting, PFS was superior in the group receiving ≥1000 mg/m^2^ cumulative dose of bendamustine compared with those receiving 800–999 mg/m^2^ (*p* = 0.04), even when adjusted for patient age and fitness. In the relapsed cohort, those who received doses of <600 mg/m^2^ had poorer PFS outcomes compared with those who received ≥600 mg/m^2^ (*p* = 0.02). Therefore, when feasible full dose BR should be considered in WM patients.

**Bruton Tyrosine Kinase inhibitors**. The introduction of BTKi have been an important treatment option in WM, in the first line treatment as well. In a long term FU study of ibrutinib monotherapy at 420 mg orally daily, in treatment naïve patients (*n* = 30), the 4‐year PFS rate was 76% and 11 patients had discontinued treatment after median FU of 50 months. Atrial fibrillation occurred in 6 patients.^53^ Since flat dosing treatment regimens are not a fit for all solution, dose reductions ibrutinib may be necessary. An analysis from a cohort of 353 ibrutinib treated patients identified 96 (27%) patients requiring a dose reduction due to adverse events (AE) such as musculoskeletal, gastrointestinal or dermatologic symptoms, cardiac events, and cytopenias.^54^ Most AEs improved after dose reduction and most patients maintained their remission status. Another option for patients experiencing AEs is switching of the BKTi. A prospective study examined 67 patients with previously treated B‐cell malignancies including WM, who were intolerant of ibrutinib or acalabrutinib or both. Most intolerance events resolved when patients switched to zanubrutinib.^55^

Zanubrutinib is an approved BTKi for patients with WM. Zanubrutinib was compared to ibrutinib in a large, open label randomized phase 3 trial in 201 patients with mostly relapsed WM.^56^ Efficacy was comparable between the two BTKi but AEs were significantly less when using zanubrutinib, with lower rates of atrial fibrillation (8% vs. 25%) and hypertension (15% vs. 26%). In an update at the IWWM‐11 meeting, with 45 months median FU, fewer patients receiving zanubrutinib had an AE leading to treatment discontinuation (9% vs. 20%) or dose reductions (14% vs. 23%). Zanubrutinib provided faster and deeper responses in patients with *MYD88* ^MUT^ *CXCR4* ^MUT,^ and responses to zanubrutinib in a separate cohort of patients with *MYD88* ^WT^ continued to deepen over time. Of note, differing responses with BTKi in patients with *MYD88* ^*L265P*^ and *MYD88* ^*WT*^ can be attributed to great variability in mutation analysis methods used in the clinical trials, highlighting the necessity of a consistent method and sensitivity of MYD88 analysis. However, until now, no difference in PFS or OS between zanubrutinib and ibrutinib has been documented.

There is now sufficient long‐term data in the first‐line and relapsed setting to suggest that BTKi should be preferentially used in patients with significant co‐morbidities or frailty predicting increased risk of chemotherapy‐associated toxicity. However, prospective randomized clinical trials comparing rituximab‐chemotherapy with BTKi, including patient reported outcomes and health economic analyses are needed to chart the role of both approaches in the modern era.

Although BKTi are active in WM, BTKi resistance has been described due to acquired BTK mutations. Therefore, non‐covalent BTKi such as pirtobrutinib that bind an alternate site have been studied. A phase 1/2 study in patients with B‐cell malignancies including 26 patients with relapsed WM found pirtobrutinib to be safe and active, despite previous treatment with covalent BTK inhibitors. Neutropenia (20%) was the most frequent occurring grade ≥3 AE and low rates of grade ≥3 AEs of hypertension (3%), hemorrhage (2%), and atrial fibrillation/flutter (1%) were seen with pirtobrutinib.^57^

**Proteasome inhibition**. Proteasome inhibitors have been used in the treatment of WM for over 10 years, mostly studied in small, single arm, prospective trials, in both newly diagnosed and relapsed patients. Overall response rates (ORR) in these trials varied between 75% and 89% and median PFS between 6.6 and 43 months.^58^, ^59^, ^60^, ^61^, ^62^, ^63^

A recent retrospective analysis of 6 United Kingdom centers identified 41 patients who received 44 bortezomib‐containing regimens (*n* = 12 frontline, *n* = 32 relapse). The ORR was 88%; 2‐years OS and PFS were 90% and 76%, respectively.^64^ Bortezomib should be considered as a treatment modality particularly in those who are refractory to BTKi. The high incidence of peripheral neuropathy is however an issue. A recent analysis of the ECWM phase II trial comparison of Bortezomib‐DRC versus DRC in first‐line treatment of 202 patients showed that adding bortezomib led to faster and deeper responses.^65^ However, there was increased neurotoxicity, and concern for increased infections, without a difference in PFS between the 2 regimens. In 2 separate prospective phase II trials in both newly diagnosed and relapsed/refractory WM, ixazomib was combined with rituximab and dexamethasone (IRD).^66^, ^67^ Therapy consisted of 6–8 cycles IRD followed by rituximab maintenance for 1–2 years. In total 85 patients were included and after IRD induction, ORR was 71%–96% with 14%–19% VGPR, and 37%–77% PR. After a median FU of 52 months median PFS and OS were 40 months and no deaths reported in the group of 26 newly diagnosed patients, while after a median FU of 24 months, PFS and OS were 56% and 88%, respectively in the relapsed and refractory group of 59 patients. Ixazomib compared favorably to bortezomib in terms of neuropathy since discontinuation of therapy or increase in symptom burden due to neuropathy did not occur with ixazomib.^66^

**Future**. A future option for the treatment of relapsed/refractory WM is the BCL‐2 inhibitor venetoclax. In a multicenter phase II trial, 32 patients were treated with 800 mg (escalated from 200 mg at start) for a total of 2 years. The overall, major, and VGPR rates were 84%, 81%, and 19%, respectively. The median PFS was 30 months with a sharp decline after 2 years when treatment was stopped per protocol definition. Importantly, CXCR4 mutations did not seem to affect treatment response or PFS.^68^

In **conclusion**, the diagnostic accuracy and treatment landscape of WM is improving in the recent years. The availability of several effective agents and increasing importance of patients' treatment preferences will lead to a more individualized treatment approach in WM that accounts for treatment and patient characteristics. Future trials should focus on effective combinations of novel agents with a focus on fixed duration, high efficacy and low toxicity.

## CONFLICT OF INTEREST STATEMENT

Karima Amaador; none. Catherine Thieblemont: honoraria from Roche, Amgen, Janssen, Celgene, Gilead Science/Kyte Beigene; consulting/advisory role for Roche, Gilead Sciences, Janssen, Celgene, Novartis, Beigene, BMS/Celgene research funding and travel, accommodations, expenses from Roche, Novartis. Judith Trotman; Research funding from Roche, Takeda, Beigene, Cellectar, Janssen, BM. Monique Minnema; Consulting for Janssen Cilag, GSK, CDR life, Pfizer. Speakers Bureau Janssen Cilag, BMS, WebMD, Research funding Beigene

### PEER REVIEW

The peer review history for this article is available at https://www.webofscience.com/api/gateway/wos/peer-review/10.1002/hon.3210.

## Data Availability

Data sharing is not applicable to this article as no new data were created or analyzed in this study.
